# MOCA for Integrated Analysis of Gene Expression and Genetic Variation in Single Cells

**DOI:** 10.3389/fgene.2022.831040

**Published:** 2022-03-31

**Authors:** Jared Huzar, Hannah Kim, Sudhir Kumar, Sayaka Miura

**Affiliations:** ^1^ Institute for Genomics and Evolutionary Medicine, Temple University, Philadelphia, PA, United States; ^2^ Department of Biology, Temple University, Philadelphia, PA, United States; ^3^ Center for Excellence in Genomic Medicine and Research, King Abdulaziz University, Jeddah, Saudi Arabia

**Keywords:** tumor evolution, single-cell RNA sequencing, gene expression trajectory, cellular phylogeny, multi-omics analyses

## Abstract

In cancer, somatic mutations occur continuously, causing cell populations to evolve. These somatic mutations result in the evolution of cellular gene expression patterns that can also change due to epigenetic modifications and environmental changes. By exploring the concordance of gene expression changes with molecular evolutionary trajectories of cells, we can examine the role of somatic variation on the evolution of gene expression patterns. We present Multi-Omics Concordance Analysis (MOCA) software to jointly analyze gene expressions and genetic variations from single-cell RNA sequencing profiles. MOCA outputs cells and genes showing convergent and divergent gene expression patterns in functional genomics.

## 1 Introduction

Cancer cells in tumors harbor extensive genetic and gene expression heterogeneity (Gray and Collins, 2000; Russnes et al., 2011; Zhang et al., 2013; Dentro et al., 2021). Genetic heterogeneity is due to somatic mutations that create new cell genotypes that experience neutral, adaptive, or purifying selection (Merlo et al., 2006; Gerlinger et al., 2012; Greaves and Maley, 2012; Davis et al., 2017; McGranahan and Swanton, 2017). Somatic mutations can also cause changes in gene expression, with epigenetic modifications and environmental factors also contributing to the heterogeneity of gene expressions (Burrell et al., 2013; Deshmukh and Saini, 2020; Mbemi et al., 2020). Single-cell RNA sequencing (scRNA-seq) is now widely used to interrogate the expression of genes in large numbers of cells (Shalek et al., 2013; Trapnell et al., 2014; Baron et al., 2016; Haque et al., 2017). Also, it has begun to be used to assess genetic variation and reconstruct cellular phylogeny (Ramazzotti et al., 2020; Zhou et al., 2020; Moravec et al., 2021), albeit with high rates of missing data and false-negative detection of mutant variants (Fasterius et al., 2019; Liu et al., 2019). Therefore, in principle, the evolution of cell expression and genetic architecture can be inferred from scRNA-seq.

We have developed a tool for Multi-Omics Concordance Analysis (MOCA) of scRNA-seq datasets to test whether gene expression and genetic evolution show concordance patterns. MOCA classifies cells based on somatic variants (genetic ancestry) and gene expression profiles (expression state), captured in scRNA-seq. MOCA analysis reveals cells whose expression type matches their ancestry relationships (divergent expression evolution) and those in the same expression state despite being from genetically distinct ancestry (convergent expression evolution). We present multiple example data analyses to validate MOCA and demonstrate its usefulness. These applications suggest that MOCA can illuminate links between phenotypic (e.g., gene expression patterns) and genetic changes useful for understanding cancer progression, therapy resistance, and tumorigenesis.

## 2 Methods

### 2.1 Overview of MOCA Tools

**FIGURE 1:**
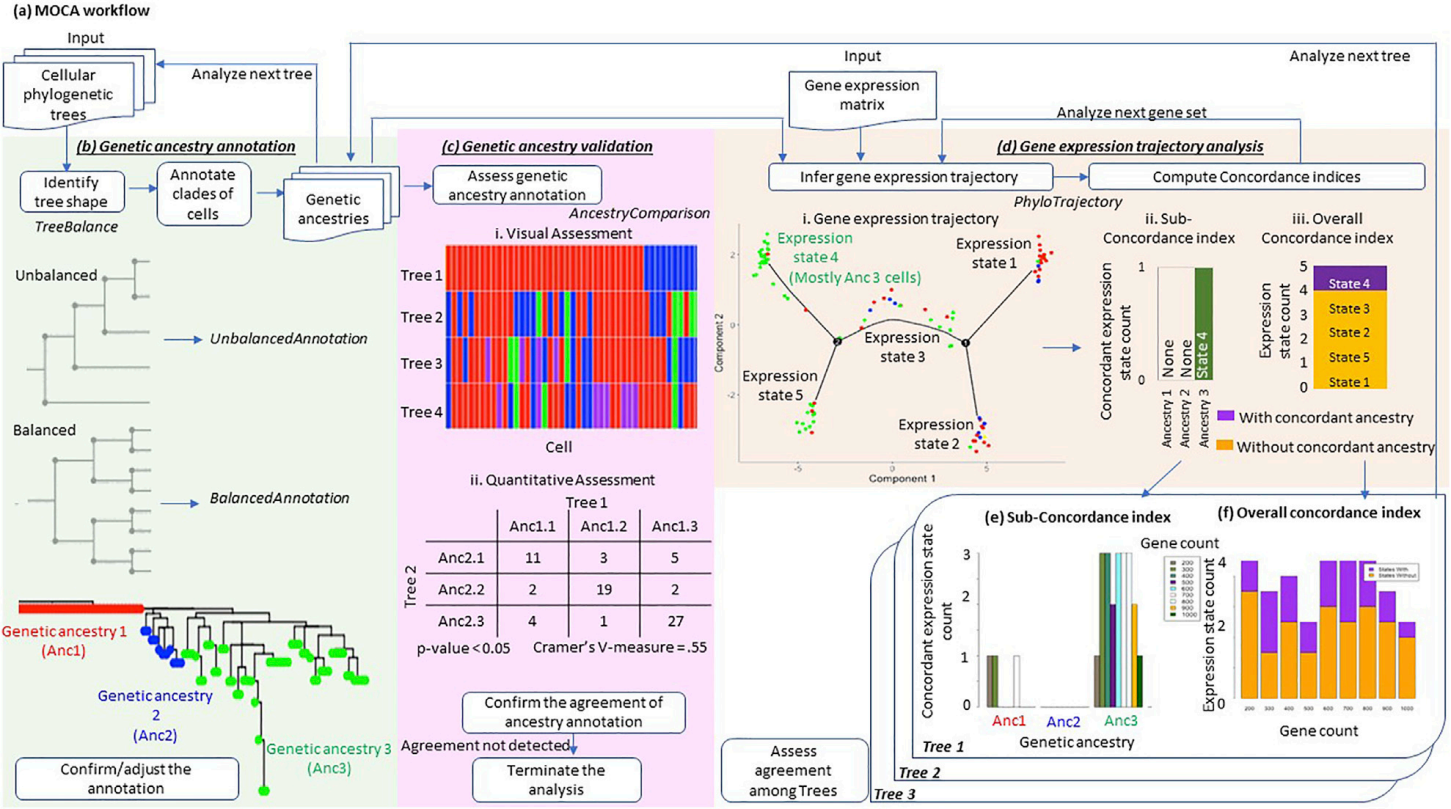
Overview of MOCA. **(A)** MOCA workflow. MOCA analyzes cellular genetic variations and gene expression profiles from scRNA-seq data. **(B)** MOCA takes as input a phylogenetic tree if the genetic ancestry of each cell is not provided. If the type of tree shape (balanced or unbalanced) is unclear, MOCA’s *TreeBalance* function first suggests the tree’s shape. Based on the tree shape, MOCA suggests using either the *BalancedAnnotation* or the *UnbalancedAnnotation* function to identify groups of genetically similar cells, which are defined as genetic ancestries. The phylogeny was inferred by BEAM analysis of 139 variants in the MGH26 data. **(C)** MOCA’s *AncestryComparison* function visualizes and quantifies the relationship between inferred genetic ancestry annotations among different phylogenies. For each pair of trees (genetic ancestry annotations), Cramer’s V effective size together with *p*-value is produced. **(D)** Using the genetic ancestries together with the gene expression matrix (input), MOCA’s *PhyloTrajectory* function infers the expression trajectory. From the inferred trajectory, *PhyloTrajectory* calculates the *Sub-concordance index* (*SCI*) for each genetic ancestry and *Overall concordance index* (*OCI*). The *SCI index* is the count of expression states which are largely unique to a given genetic ancestry, e.g., >80% of cells. The *OCI* is the ratio of total expression states that are largely unique to any single ancestry compared to all the expression states identified. **(E)** The *SCI index* for each genetic ancestry for different numbers of genes, 200–1,000. **(F)** The *OCI* of the tumor across gene sets, 200–1,000. These indices are produced for each tree.

MOCA conducts three distinct analyses: 1) genetic ancestry annotation from cellular phylogenies, 2) assessment of genetic ancestry annotation, and 3) gene expression trajectory analysis in the context of the genetic ancestry (Figure 1A).

#### 2.1.1 Genetic Ancestry Annotation Tools

MOCA identifies cells with similar or identical genotypes in the phylogeny (Figure 1B), given an inferred cellular phylogeny. MOCA presents two approaches to develop groups of cells (ancestry type): one for ladder-like phylogenies (*UnbalancedAnnotation* function) and the other for balanced-shape phylogenies (*BalancedAnnotation* function), following Paradis et al. (2004). Both functions circumscribe groups of cells such that genetic distances between cells within groups are smaller than between groups (see *BalancedAnnotation and UnbalancedAnnotation* for the detail). Cells within the same group are assigned to a genetic ancestry type. If users need guidance in selecting which annotation function to use, MOCA provides the *TreeBalance* function, which analyzes the shape of a given phylogeny (Bortolussi et al., 2006).

To empower users to confirm the annotation visually, MOCA outputs cell phylogeny with the estimated genetic ancestries mapped (e.g., Figure 1B). MOCA also produces annotation files for manual editing and expert refinement. Of course, alternatively, other cell clustering methods such as SCG (Roth et al., 2016) may be used to directly annotate genetic ancestry from observed single-cell sequences without reconstructing cellular phylogenies. In this case, genetic ancestry may be assigned to cells that are in the cell group (clone) by the chosen method.

#### 2.1.2 Genetic Ancestry Validation Tools

When there is a large amount of missing data and false-negative detection of mutant variants, which is not uncommon in scRNA-seq data, the inferred cell phylogeny may not allow reliable genetic ancestry annotation. Therefore, we need to validate genetic ancestry annotation, if possible. MOCA compares genetic ancestry annotations produced by two and more than two computational methods and presents a visualization of the agreement of ancestry annotation between phylogenies together with statistical measures, i.e., Cramer’s V effect size and *p*-value (see *AncestryComparison* for details) (Figure 1C). We may consider genetic ancestry annotations reliable if the use of phylogenies from different approaches produces consistent results. We also suggest that the downstream gene expression trajectory analysis should be performed only if there is concordance among ancestry annotations from multiple phylogenies.

#### 2.1.3 Gene Expression Trajectory Analysis Tools

MOCA’s *PhyloTrajectory* function analyzes the gene expression matrix produced by scRNA-seq data analysis. *PhyloTrajectory* uses Monocle (Qiu et al., 2017; Trapnell et al., 2014) to reconstruct a gene expression trajectory, which also takes information on evolutionary clusters (genetic ancestries) as input (Figure 1D). In Monocle, the non-cancerous (normal) cell is set to be the origin, i.e., it receives the pseudotime equal to zero. Genes differentially expressed between given genetic ancestries are identified (Yee, 1996), and a gene expression trajectory using these genes is produced together with cell by cell annotation of the expression state. Cells with similar gene expression patterns receive the same expression state.

In the next step, the *PhyloTrajectory* function assesses whether cells with the same genetic ancestry tend to have the same expression state. Two measures are made available, one for each genetic ancestry (*Sub-concordance index; SCI*) and the other for the whole tumor (*Overall concordance index; OCI*). *SCI* is calculated for each genetic ancestry. It is the count of expression states that are uniquely composed of cells belonging to a given genetic ancestry. To further describe *SCI* calculation, we use an example gene expression trajectory shown in Figure 1D. For example, many cells from ancestry 3 have gene expression state 4, which rarely has cells from the other ancestries. On the other hand, the other expression states contain cells from various ancestries. In this case, the *SCI* of ancestry 3 is one. The minimum value of *SCI* is zero (e.g., ancestry 1 and 2 in Figure 1D), indicating cells from the given ancestry do not have a unique gene expression pattern. A count greater than one indicates that cells from the same ancestry can be further subclassified into distinct groups by the similarity of gene expression patterns. Second, *OCI* is the ratio of total expression states that are unique to single ancestries in the data (Figure 1D). *OCI* indicates the proportion of new expression states evolving within cancer cell populations of the same genetic ancestry.

The above analysis is repeated over the desired range of gene counts (see *PhyloTrajectory* for details), and the concordance indices are summarized across the gene counts (Figures 1E,F). When a limited number of genes’ expression patterns are altered along with genetic changes, concordance between genetic ancestry and gene expression states would be detected only when a small number of genes are used. This is because the use of a greater number of genes will dilute the biological signal by including the genes that do not follow that trend.

In addition, *PhyloTrajectory* performs the above analysis for each genetic ancestry annotation from different phylogenies, which is important to consider the uncertainty of genetic ancestry annotation. All inferences are visualized so that a researcher can assess consistency when slightly (or more) different genetic ancestry annotations are used. Such analyses evaluate the robustness of the concordance seen between genetic ancestry and gene expression state annotations. Users can also perform the gene expression trajectory analysis using a consensus genetic ancestry annotation produced using the *AncestryComparison* function (see *AncestryComparison*) to evaluate the inference further.

### 2.2 MOCA Functions

#### 2.2.1 TreeBalance

For a given phylogeny, the *TreeBalance* function uses the apTreeshape method and estimates the beta value of the Beta-splitting model (Bortolussi et al., 2006). Trees with beta values less than −1.5 are designated as unbalanced trees (PDA model), while trees with beta values of greater than −1.5 are designated balanced trees (Yule trees).

#### 2.2.2 BalancedAnnotation and UnbalancedAnnotation

The *BalancedAnnotation* and *UnbalancedAnnotation* functions use tools in the ape package (Paradis et al., 2004). The *BalancedAnnotation* function iteratively identifies clades of cells (genetic ancestry) until the desired number of clades (default: 3) is determined. *BalancedAnnotation* first performs pairwise testing on all possible clades to identify two clades that have the least amount of genetic dissimilarity within each. The first two clades are also required to have the desired minimum percent of total cells (default: 0.75), while each clade has the desired minimum percent of cells (default: 0.1). Next, the largest remaining clades are identified. If two remaining clades are of equal size, then the clade with the least genetic dissimilarity is selected.

The *UnbalancedAnnotation* function partitions a given phylogeny into the desired number of ancestries (default: 3), in which each ancestry will have a similar number of cells. For each ancestry, a single clade is identified from the tree containing the nearest desired number of cells at each time. Then the next clade containing the nearest desired number of cells is searched after removing the first clade from the phylogeny. This process is repeated until the desired number of clades is determined. If there are two potential clades, the clade with the least genetic dissimilarity is selected.

#### 2.2.3 AncestryComparison

For all the trees or genetic annotation files provided, *AncestryComparison* produces a consensus annotation of genetic ancestries and returns a heatmap colored with genetic ancestry IDs, where each cell is aligned across the trees (or annotation files) together with the consensus annotation. *AncestryComparison* will also assess the annotation agreement between each pair of trees (or annotation files), including the consensus annotation, by calculating Cramer’s V effect size and *p*-value (a chi-square test). These measures are based on a contingency table of cell count for each combination of ancestry annotation (e.g., cells classified Ancestry 1.1 for a phylogeny 1 and Ancestry 2.1 for a phylogeny 2 in Figure 1C). These statistical measures are calculated for each pair of phylogenies. With Cramer’s V effect size >0.2 with *p* << 0.01, the agreement of ancestry annotations between two phylogenies may be supported.

#### 2.2.4 PhyloTrajectory

A set of expression trajectories is inferred using the desired range of gene counts for each tree (genetic ancestry annotation). By default, MOCA starts with the 200 most differentially expressed genes and then adds the next top 100 most differentially expressed genes until reaching the total of 1,000 genes that are most differentially expressed between genetic ancestries. Genes are first ranked based on the extent of their expression level differences between genetic ancestries, using a method in Monocle (Yee, 1996). For each desired number of genes (gene scale), the dataset is restricted to the top genes most differentially expressed between genetic ancestries. Monocle is used to infer the gene expression trajectory.

### 2.3 Data Collection and Analysis

We obtained a gene expression matrix (Hou et al., 2016) from the GEO database (http://www.ncbi.nlm.nih.gov/geo) (GSE65364). After removing redundant genes and genes which were not expressed, the gene expression matrix contained 14,611 genes. The raw sequence reads were obtained from the NCBI Sequence Read Archive (https://www.ncbi.nlm.nih.gov/sra) (SRP052901). We refer to this as the Hou dataset. We also obtained other filtered and normalized gene expression matrices of 5,948 genes that were previously generated, i.e., MGH26 and the MGH31 datasets (Patel et al., 2014) from the GEO database (http://www.ncbi.nlm.nih.gov/geo) (GSE57872). We obtained read sequences from the NCBI SRA (https://www.ncbi.nlm.nih.gov/sra) (PRJNA248302).

Raw read sequences from both datasets were aligned to the human reference genome sequence (hg19) using STAR (Dobin et al., 2013), and single nucleotide variants (SNVs) were called using samtools (Li, 2011). Based on non-cancerous cells reported in the original study (Patel et al., 2014), we removed SNVs when all normal cells did not have a base assignment (missing data), when base assignments were inconsistent among the normal cells, or when they were heterozygous or were different from the reference genome (potential germline heterozygosity). We also excluded low-quality cells, where >50% of SNVs were missing.

To infer cellular phylogeny, we analyzed SNV profiles using SCITE (Jahn et al., 2016) and BEAM (Miura et al., 2018). In BEAM analysis, we excluded cells that received low posterior probabilities of their refined base assignments because such cell sequences are of low quality, e.g., many sequencing errors. In SCITE analysis, we used the default false negative and false positive detection rates of mutation (0.21545 and 1.3 × 10^–5^, respectively).

## 3 Results

### 3.1 Hou Data Analysis

First, we analyzed the Hou data using MOCA. The purpose was to validate MOCA’s performance. The original study sequenced both DNA and RNA from each hepatocellular carcinoma cell using a single-cell triple omics sequencing technique (scTrio-seq) (Hou et al., 2016). We began by testing if SNVs detected from RNA sequences can produce genetic ancestries similar to those obtained from the analysis of their DNA sequences.

**FIGURE 2:**
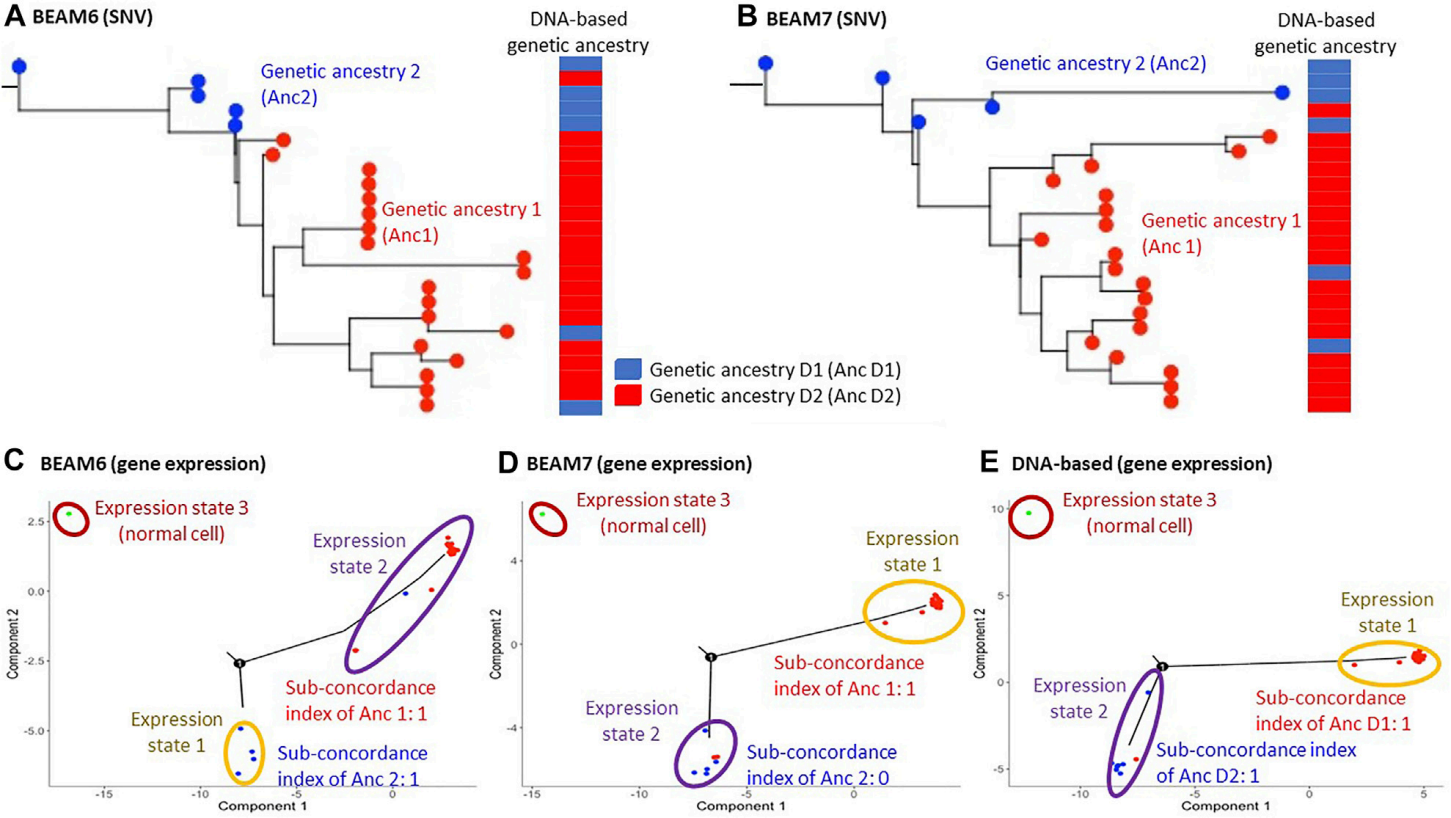
Hou data analysis. **(A,B)** Inferred phylogenies on datasets where 60% **(A)** and 70% **(B)** SNV filtering cutoffs were applied. BEAM was used for building cellular phylogeny to account for missing data and mutation calling errors. The genetic ancestry annotation obtained from the analysis of DNA data is shown next to the phylogenies. The DNA-based annotation was obtained from Hou et al. (2016). **(C–E)** The inferred expression trajectory using genetic ancestries annotated on the dataset with 60% SNV filtering cutoff tree **(C)**, 70% SNV filtering cutoff tree **(D)**, and DNA-based genetic ancestry annotation **(E)**. 1,000 most differentially expressed genes between the genetic ancestries were used in each analysis.

We prepared two datasets by filtering SNVs from RNA data with two different cutoffs of the proportion of unambiguous base assignment among cells (70% and 60%). After filtering SNVs, the total number of SNVs in the datasets were 123 and 168, respectively, which is around the minimum number required for optimum performance of the phylogeny reconstruction methods for single-cell sequencing data, e.g., BEAM (Miura et al., 2018). The inferred phylogenies are shown in Figures 2A,B. Since the original study analyzed DNA sequences and classified the cells into two groups based on the presence and absence of copy number alterations (CNAs), we mapped these classifications onto our phylogenies inferred using SNVs from RNA sequences of the same cells. Cells in the same DNA-based group clustered well on our RNA-seq phylogenies, with only 3 cells showing disagreements. This result suggested that SNVs from RNA sequences can be used for genetic ancestry annotation, which is also consistent with reports from a previous study (Moravec et al., 2021).

In addition, the original study analyzed gene expression patterns and reported that genes affected by the CNAs also showed proportional changes in their expressions (Hou et al., 2016). This result suggests that gene expression alterations followed genetic changes. Therefore, we tested if MOCA’s gene expression trajectory analysis and genetic ancestry can produce the same inference. Figures 2C,D showed the gene expression trajectories when the RNA-seq genetic ancestries were given. The concordance between genetic ancestry and gene expression annotation for both phylogenies was detected. For example, the use of the dataset with 70% SNV filtering cutoff (BEAM7 in Figure 2B) produced two gene expression states (excluding the state for the normal cell), and one of them agreed with genetic ancestry annotation (Figure 2D). Also, the use of genetic ancestry annotation from the original study (DNA-based annotation) produced good concordance between genetic and expression states annotations (Figure 2E). Therefore, these inferences were consistent with the original study, validating MOCA’s performance.

The above results were based on the expression trajectory analysis of 1,000 genes. We repeated these MOCA analyses using a smaller number of genes. Interestingly, the use of a smaller number of genes (<700 genes) did not detect concordance between genetic and gene expression annotations (Supplementary Figure S1). This is likely because the quality of gene expression data was low due to the technical limitation of scTrio-seq, i.e., the number of zero expression data was much larger than the other two datasets from scRNA-seq. Therefore, the use of a greater number of genes was necessary to detect concordance between genetic and gene expression annotations.

### 3.2 Analyses of MGH26 Datasets

**FIGURE 3:**
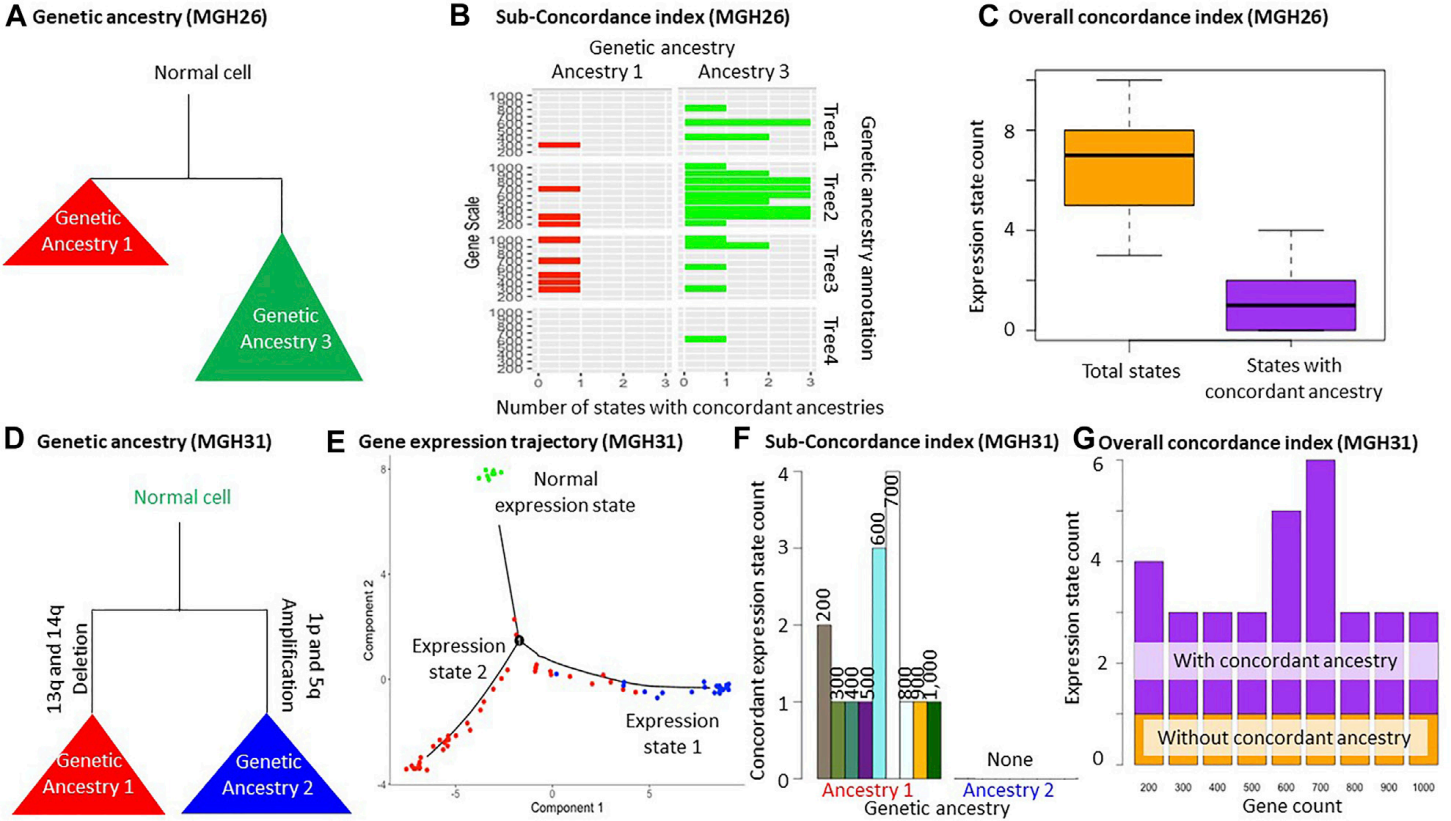
Glioblastoma tumor data analysis. **(A–C)** Analysis of MGH26 tumor data. **(A)** Schematic of genetic ancestries that are consistent across four phylogenies (Supplementary Figure S2). **(B)** *Sub-concordance index* (*SCI*) of genetic ancestry 1 and 3. **(C)** The number of all expression states and states that are unique to genetic ancestries allowing a few exceptions (>80% of cells from an ancestry share the same expression state). **(D–G)** Analysis of MGH31 tumor dataset. **(D)** Schematic of genetic ancestries. Copy number aberrations were used for genetic ancestry annotation for MGH31 data. Cells of genetic ancestry 1 contained 13q and 14q deletion, while cells of genetic ancestry 2 contained 1p and 5q amplification. **(E)** The inferred expression trajectory using the top 1,000 most differentially expressed genes between the genetic ancestries. All normal cells were predicted to have the same gene expression state. **(F)** The *Sub-concordance index* (*SCI*) of each genetic ancestry. Normal cells were excluded, which always had a concordance index equal to one. **(G)** The *Overall concordance index* (*OCI*). The expression state for normal cells was included. The MGH26 and MGH31 datasets are from Patel et al. (2014).

We also applied MOCA to two glioblastoma tumor datasets (MGH26 and MGH31). We prepared two datasets in the same fashion as the Hou data analysis for each tumor, i.e., SNV filtering with different cutoffs. Then SCITE (Jahn et al., 2016) and BEAM (Miura et al., 2018) were used for phylogenetic reconstruction. In total, we obtained 4 cell phylogenies for each tumor (two datasets analyzed by two methods) to make sure that the two trees did not accidentally show appreciable agreements in genetic ancestry annotations (Figure 1B and Supplementary Figure S2). We first present the result for the MGH26 dataset. The total numbers of SNVs in these datasets were 139 and 180. We chose to generate three genetic ancestry groups for each phylogeny using the *TreeBalance*, *UnbalancedAnnotation*, and *BalancedAnnotation* functions. Visual comparison of genetic ancestry annotation among these four phylogenies using the *AncestryComparison* function showed that two genetic groups (ancestry 1 and 3) were largely consistent among phylogenies (Figures 1C, 3A). Even when we attempted to make three groups, only two groups of cells could be reliably distinguished. So, we did not consider any more groups. Next, we statistically confirmed the visual agreement between the two ancestries across phylogenies, i.e., all pairs of annotations resulted in Cramer’s V effect sizes ranging from 0.38 to 0.67 (*p* << 0.01). Cramer’s V effect size higher than 0.2 implies a moderate association between annotations (Supplementary Figure S3A). We also tried Rand Index but found it overly conservative in our exploratory analyses (results not shown). Therefore, we did not implement Rand Index in MOCA. Last, our visual inspection of the inferred cellular phylogenies did not find other possible genetic ancestry annotations, so we explored only two ancestries in the downstream gene expression trajectory analysis.

We performed gene expression trajectory analysis using these genetic ancestry annotations and 200–1,000 genes using the *PhyloTrajectory* function (Figure 1D and Supplementary Figure S2). Based on the *Overall concordance index*, the number of expression states was often more than twice the number of genetic ancestries. One or two states were generally unique to certain genetic ancestries (Figures 3B,C). This pattern indicates that some cells from the same genetic ancestry evolved distinct gene expression patterns, while gene expression patterns converged for some cells from different genetic ancestries. This inference was consistently obtained in analyses with a small and large number of genes. Also, the inferences were consistent across slightly different ancestry annotations from different phylogenies, demonstrating their robustness (Figures 3B,C and Supplementary Figure S2).

### 3.3 MGH31 Data Analysis

In contrast to the Hou and MGH26 datasets, genetic ancestry annotation did not agree well between inferred phylogenies in the MGH31 data analysis (*p* = 0.03–0.1; Supplementary Figure S3B). This is likely because of the presence of large-scale deletions that complicate phylogenetic inference due to the loss of variants. For example, SCITE assumes a lack of deletions (Jahn et al., 2016). In this case, the use of genetic ancestry annotation based on copy number alteration may be more appropriate (Figure 3D). Therefore, we performed a *PhyloTrajectory* analysis with two genetic ancestry groups based on the similarity of copy number alterations, which were previously reported for this dataset (Serin Harmanci et al., 2020). From *PhyloTrajectory*, we identified one expression state unique to ancestry 1, which harbors 13q and 14q deletions, and another state for the normal cells (Figure 3E). Genetic ancestry 2, consisting of cells that contain the 1p and 5q amplification, had no unique expression states for the ancestry because the state that contained ancestry 2 was also shared by some cells from ancestry 1 (Figure 3E). A similar result was consistently produced in analyses with different sets of genes (Figures 3F,G).

## 4 Discussion

MOCA is a collection of tools for exploring tumor evolution using genetic variation and gene expression profiles from the same cells. MOCA is designed for scRNA-seq data that enables the profiling of genetic variation and gene expressions of cells. We have demonstrated its potential through analyses of two scRNA-seq glioblastoma datasets. This study also validated MOCA’s performance on a dataset with known cellular populations and expression alterations.

But, it is important to note some caveats. Firstly, MOCA tests the reliability of genetic ancestry annotation by assessing the similarity of clusterings obtained from different phylogenies using distinct methods. These methods have unique strengths as well as weaknesses. For example, SCITE may not perform well for datasets with a large number of SNVs (>1,000), and the selection of BEAM is not recommended for datasets with a large number of cells (>2,000) (Chen et al., 2020). Therefore, methods need to be cautiously selected for a given dataset. Also, in analyzing empirical datasets, it is not always possible to know about the optimal approach. When the most appropriate way is in doubt, we suggest applying multiple methods and looking for consistent patterns.

Secondly, the number of genetic ancestries to consider in MOCA analysis is subjective. Users can choose to have a small or large number of clades by setting parameters (see *BalancedAnnotation and UnbalancedAnnotation*). When a dataset contains many cells, a large number of cell groups may be defined. The same may be possible if scRNA-seq datasets cover many SNVs. For such datasets, branch supports obtained by Felsenstein’s bootstrap procedure for molecular phylogenies (Felsenstein, 1985; Nei and Kumar, 2000) may be useful to identify reliable clades for genetic ancestries.

In conclusion, MOCA provides tools to assess the reliability of genetic ancestry annotation from phylogenies inferred using noisy genetic data (i.e., SNVs detected in scRNA-seq data). MOCA also produces simple measures of concordance between genetic ancestries and gene expression state annotations, which would guide users to explore divergent and convergent evolution of gene expression patterns. MOCA is written in R and is available from https://github.com/SayakaMiura/MOCA.

## Data Availability

The datasets presented in this study can be found at https://github.com/SayakaMiura/MOCA.
