# Supplementary material for: MOCA for Integrated Analysis of Gene Expression and Genetic Variation in Single Cells
Source: Front Genet. 2022 Mar 31;13:831040. doi: 10.3389/fgene.2022.831040 (PMC9009314; doi:10.3389/fgene.2022.831040)
Supplement: Supplementary file 1 [file DataSheet1.PDF]

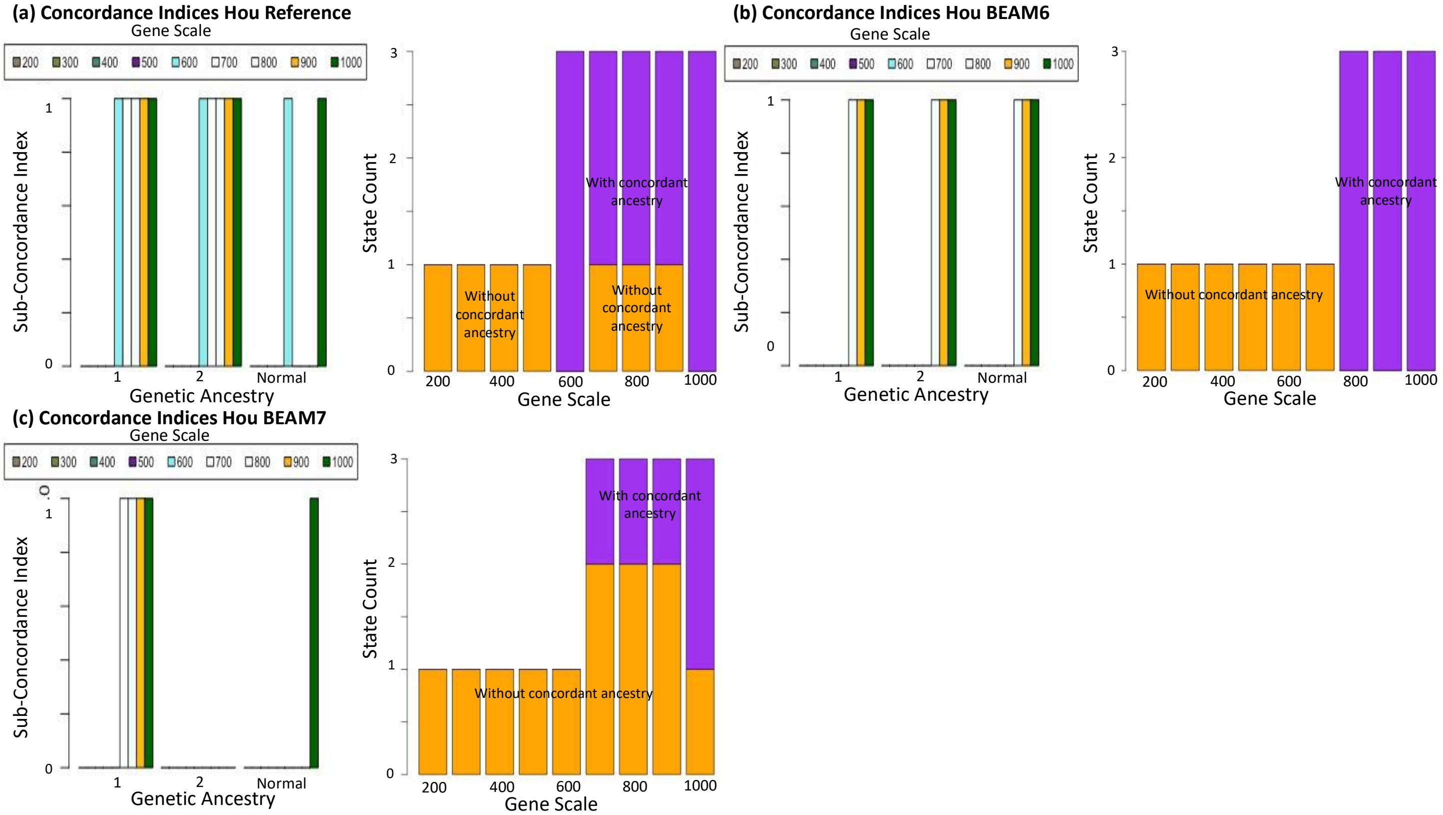


**Supplementary Figure 1. Concordance indices from Hou data analysis.** Gene expression trajectories were inferred by providing (**a**) genetic ancestry annotation using DNA sequences and (**b** and **c**) annotations using RNA sequences. SNV filtering cutoffs of 60% (**b**) and 70% (**c**) were used. Genetic ancestry annotations were presented in **Figures. 2a** and **2b**.

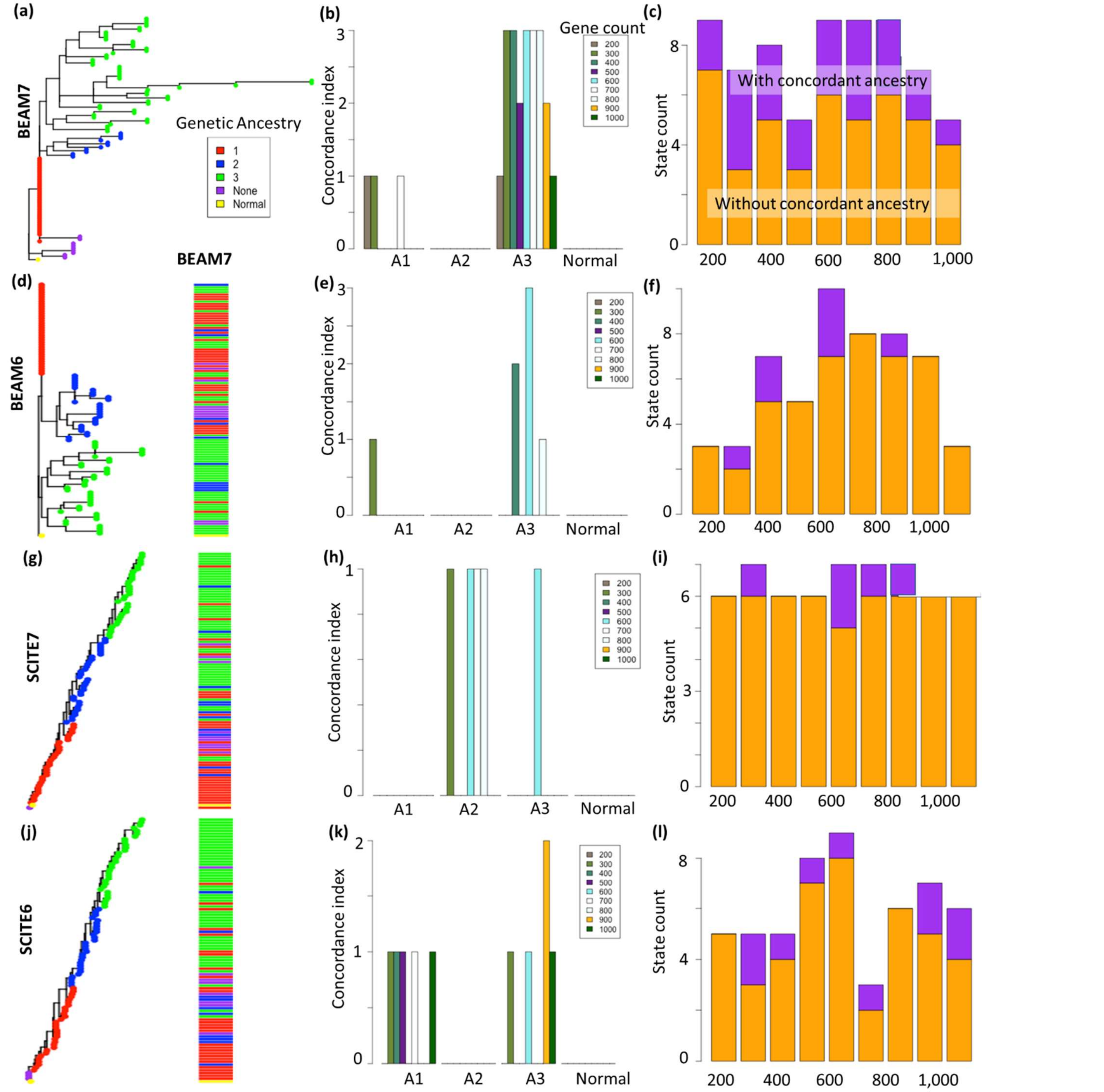


**Supplementary Figure 2. MGH26 data analysis with MOCA.** Four phylogenies were reconstructed using different computational tools (BEAM and SCITE) and SNV filtering cutoffs (60% and 70%). **(a-c)** The phylogeny was reconstructed using BEAM on the dataset, where 70% SNV filtering cutoff was applied (BEAM7). (**a**) The inferred phylogeny. (**b**) The *Sub-concordance index* (*SCI*) for each ancestry. (**c**) The *Overall concordance index* (*OCI*). These indices were calculated using expression trajectories that were inferred from 200 to 1,000 genes. **(d-f)** The results using BEAM on a dataset with a 60% SNV filtering cutoff, **(g-i)** SCITE with a 70% SNV filtering cutoff, and **(j-l)** SCITE with a 60% SNV filtering cutoff. (**d**, **g**, and **j**) Cellular phylogenies. The genetic ancestry annotation using BEAM with 70% SNV filtering cutoff (BEAM7) is mapped onto the phylogeny via the heatmap.

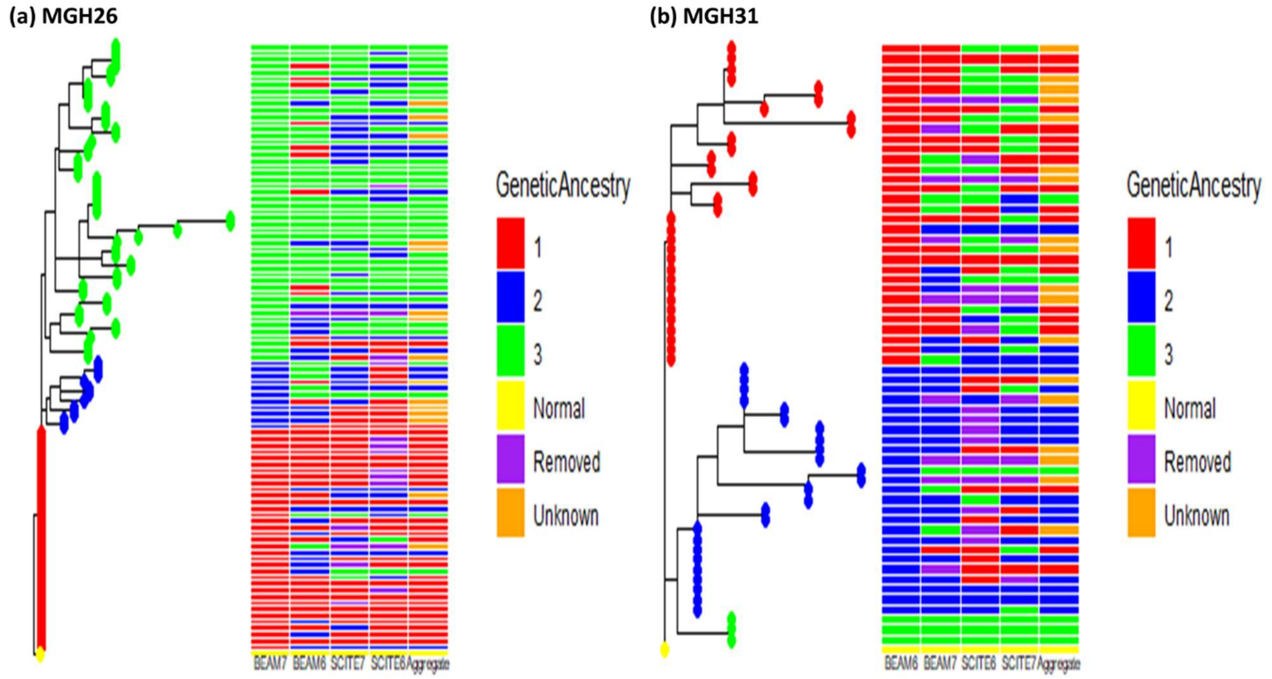


**(c) Quantitative Ancestry Assessment (MGH26)**

| | BEAM7 | BEAM6 | SCITE7 | SCITE6 | Aggregate |
|---|---|---|---|---|---|
| BEAM7 | | .381 | .584 | .478 | .597 |
| BEAM6 | <<0.01 | | .441 | .458 | .582 |
| SCITE7 | <<0.01 | <<0.01 | | .673 | .773 |
| SCITE6 | <<0.01 | <<0.01 | <<0.01 | | .867 |
| Aggregate | <<0.01 | <<0.01 | <<0.01 | <<0.01 | |

Cramer's V-measure

P-Value

**(d) Quantitative Ancestry Assessment (MGH31)**

| | BEAM7 | BEAM6 | SCITE7 | SCITE6 | Aggregate |
|---|---|---|---|---|---|
| BEAM7 | | .553 | .335 | .327 | .662 |
| BEAM6 | <<0.01 | | .361 | .394 | .755 |
| SCITE7 | .047 | .030 | | .303 | .615 |
| SCITE6 | .028 | >0.1 | .103 | | .531 |
| Aggregate | <<0.01 | <<0.01 | <<0.01 | <<0.01 | |

Cramer's V-measure

P-Value

**Supplementary Figure 3. Genetic ancestry annotation comparison.** Four phylogenies were reconstructed for both the MGH26 (**a**) and MGH31 (**b**) datasets. Rows of each heatmap represent cells in their respective position in the phylogeny reconstructed. Each column represents the genetic ancestries identified from a phylogeny inferred using a different combination of reconstruction method (BEAM and SCITE) and SNV filtering cutoff (60% and 70%). The final column represents a consensus annotation (an aggregated annotation). A cell is assigned to whichever genetic ancestry it is most commonly assigned to across all four phylogenies. **(a)** Cells are ordered based on their respective position in the phylogeny reconstructed using BEAM on the dataset with a 70% SNV filtering cutoff. Ancestries 1 and 3 largely agree across both cutoffs and reconstruction methods. **(b)** Rows of the heatmap are ordered based on the cells' positions in the phylogeny reconstructed using BEAM on the dataset with a 60% SNV filtering cutoff. Across cutoffs and reconstruction methods there show little agreement in the genetic ancestry annotation. **(c and d)** The significance of the relationship (agreement) between each pair of genetic ancestry annotations is measured using a p-value from a chi-square test, denoted by the red text, and Cramer's V-measure of association (effect size), denoted in the black text.
